# Electrochemical Biosensors for the Detection of SARS-CoV-2 and Other Viruses

**DOI:** 10.3390/mi12020174

**Published:** 2021-02-10

**Authors:** Saim Imran, Soha Ahmadi, Kagan Kerman

**Affiliations:** 1 Department of Physical and Environmental Sciences, University of Toronto Scarborough, 1265 Military Trail, Toronto, ON M1C 1A4, Canada; saim.imran@mail.utoronto.ca (S.I.); soha.ahmadi@mail.utoronto.ca (S.A.); 2 Department of Chemistry, University of Toronto, 80 St. George Street, Toronto, ON M5S 3H6, Canada

**Keywords:** electrochemical biosensor, COVID-19, SARS-CoV-2, human immunodeficiency virus, Zika virus, Ebola virus, influenza virus, viral detection, point-of-care tool, diagnostic tools

## Abstract

The last few decades have been plagued by viral outbreaks that present some of the biggest challenges to public safety. The current coronavirus (COVID-19) disease pandemic has exponentiated these concerns. Increased research on diagnostic tools is currently being implemented in order to assist with rapid identification of the virus, as mass diagnosis and containment is the best way to prevent the outbreak of the virus. Accordingly, there is a growing urgency to establish a point-of-care device for the rapid detection of coronavirus to prevent subsequent spread. This device needs to be sensitive, selective, and exhibit rapid diagnostic capabilities. Electrochemical biosensors have demonstrated these traits and, hence, serve as promising candidates for the detection of viruses. This review summarizes the designs and features of electrochemical biosensors developed for some past and current pandemic or epidemic viruses, including influenza, HIV, Ebola, and Zika. Alongside the design, this review also discusses the detection principles, fabrication techniques, and applications of the biosensors. Finally, research and perspective of biosensors as potential detection tools for the rapid identification of SARS-CoV-2 is discussed.

## 1. Introduction

Severe acute respiratory syndrome coronavirus-2 (SARS-CoV-2) emerged from Hubei province, China in late 2019, causing the coronavirus disease (COVID-19). On 11 March 2020, the World Health Organization (WHO) declared the COVID-19 pandemic and, since then, it has gone on to infect more than 20 million people worldwide, with over 800,000 deaths. Its effects have rippled into the economy, with fears of possible financial collapse and recessions on the horizon [1]. Viral infection outbreaks are a great threat to not just the public health sector but also to the global economy. Some of the most recent outbreaks of international concern include the Influenza (H1N1) pandemic in 2009–2010, HIV global epidemic (on going), Ebola outbreak and epidemic (2014–2020), Zika epidemic 2015–2016, and the current COVID-19 pandemic [2,3,4,5,6]. Viruses that lead to an outbreak often have no vaccine or any form of treatment against them. Therefore, the method of control is through mass testing to identify cases and enable contact tracing and containment [3,4,7]. As such, a rapid, sensitive, and selective diagnosis can help to curb the rates of infection and slow down spread, thus providing enough time for the development of vaccines or treatments to control the virus. Current conventional techniques, such as polymerase chain reaction (PCR), enzyme-linked immunosorbent assay (ELISA), virus culture, and western blotting-based tests, have been used in diagnostics [8]. However, these techniques are not suitable for rapid on-site analysis, as they require complex and expensive laboratory equipment and trained personnel. They are also time-consuming, and they may not be insensitive enough for early diagnosis [9,10]. These factors increase time-to-answer and the costs of the test, as well as reduce the quality of patient care, which makes it harder to employ mass testing, especially for pandemics. Accordingly, there is a need for a rapid, sensitive, and accurate point-of-care (POC) tool, which can be employed at public places or at home. Biosensors have the capability to fulfill these traits, which makes their development worth considering.

**Figure 1:**
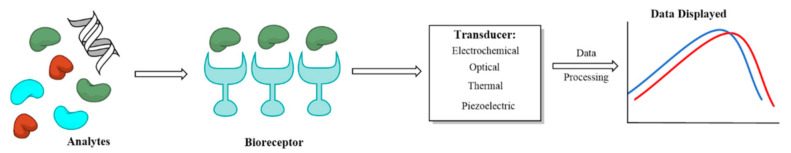
General schematic of a biosensor. Different possible bioreceptors and transducers have been shown. Adapted from Sawant et al. [14] with permission from Elsevier.

A biosensor is an analytical device that detects the biological reaction by measuring the outputs signal, which is proportional to the concentration of the analyte [11,12,13,14]. They are composed of three main parts: a bioreceptor, a transducer, and a reader device (Figure 1). The bioreceptor is a biological receptor that binds to, or catalyzes a reaction for, a specific target analyte in order to produce a detectable signal. The lifetime of a biosensor, storage (shelf-life), and/or operation time are some of the important features that may limit its commercialization. The lifetime of the biosensors is mainly dependent on the stability of their bioreceptors and the storage/operation conditions, which can be a few days to weeks [12]. However, it is expected that the commercial biosensors have the shelf-time of 6–12 months. Common bioreceptor/analyte systems are enzyme/substrate, antibody/antigen, and complementary nucleic acids, which has either a bioaffinity or biocatalytic role, depending on the specific analyte and bioreceptor. Transducers convert the bioreceptor-analyte interaction into a detectable signal, where biosensors can be classified as electrochemical, optical, thermal, or piezoelectric, depending on their transducer (Figure 1). For electrochemical biosensors, this is an electrical signal that can be detected and displayed. Several electrochemical biosensors have been developed in the last decade to detect proteins, cancer biomarkers, viruses, and bacteria. Immobilized antibodies, antigens, and nucleic acids were the most common bioreceptors used to detect viruses [15,16]. Clark and Lyons proposed the first electrochemical biosensor to detect glucose concentration in 1962, which was based on the enzyme glucose oxidase [17]. They entrapped a thin layer of glucose oxidase within a glucose permeable membrane on an oxygen electrode, and the amount of oxygen consumed determined the amount of glucose present to convey a signal. Many more biosensors have been designed since then with significant improvements being made, such as Adam Heller, who improved the detection of glucose and patented many designs on electrochemical biosensors [18,19]. Hallmarks of great biosensors are those that are highly sensitive, specific, and cost-effective. While many different approaches have been taken to designing biosensors from optical to piezoelectric to microbalance, among them, electrochemical biosensors are superior because (i) the production of microelectronic circuits for electrochemical biosensors is cost-effective and they can be easily connected to a normal electronic read-out; (ii) they are robust and user-friendly; (iii) they can be easily miniaturized and mounted on the microfluidic devices; and, (iv) they follow green chemistry principles due to their low sample/waste size [13,16]. However, like other biosensors, several aspects need to be considered for developing the electrochemical biosensors. Materials that are chosen to make biosensors can dictate the sensitive, specific, and robustness of the biosensors, as the materials must demonstrate biocompatibility with the bioreceptors to allow them to exhibit activity. Bioreceptors are also sensitive to external conditions (e.g., pH, ionic strength, temperature); hence, the immobilization matrix needs to be capable of protecting it, but still allowing for analyte to reach the bioreceptor. Improvements in immobilization, and materials, such as conducting polymers and nanoparticles, have greatly enhanced the biocompatibility and durability of biosensors, increasing their sensitivity and detection [20,21,22,23,24,25]. Advances in nanotechnology within the last few decades have resulted in major improvements in electrochemical biosensing making them simple and efficient tools to measure the concentration of analytes and the detection of pathogens. For example, Pingarrón et al. [26] summarized the improvements made while using gold nanoparticles (AuNPs), which allowed for increased sensitivity and selectivity. Additionally, developments in DNA and genome detection have also been done to make biosensors more attractive and efficient to apply in viral genome detection [27,28,29,30,31,32]. Moreover, further work has been done to miniaturize biosensors and make them portable, cost-effective, and reduce the sample size [30,33,34,35]. These improvements have made electrochemical biosensors a keystone for developing POC tools. The goal of this review is to explore recent developments in electrochemical biosensors that are designed for specific detection of full virus, viral protein, or antibodies against viral antigens for viruses that caused recent or ongoing pandemic and epidemics: influenza, HIV, Ebola, Zika, and SARS-CoV-2. Given the large number of publications in this field, each section focuses on a specific virus and electrochemical techniques that are associated with its detection in the last five years. 

## 2. Viruses Overview

Viruses are obligate intracellular parasites; hence, they need to enter a cell to be able to replicate. The basic structure of a virus consists of a genome (either DNA or RNA) that is surrounded by a protein capsid and, in the case for most animal viruses, a lipid envelope surrounding the capsid [36,37]. Entry into a cell is crucial for viruses because viruses can only replicate in the intracellular space. Hence, viral proteins are commonly expressed along the envelope, which help to recognize and bind to certain cells. Influenza contains hemagglutinin (HA) and neuraminidase (NA) as its surface proteins, which help with attachment to sialic acid residues on some mammalian cells [38]. On the other hand, HIV contains envelope proteins that recognize CD4 receptors that are expressed on T-cells, allowing for them to specifically infect T-cells [39,40]. The type of cells that a virus affects determines the type of disease that they cause and where the virus may be found. Severe acute respiratory syndrome coronavirus 2 (SARS-CoV-2) binds to Angiotensin converting enzyme 2 (ACE2) via its spike protein and, since this ACE2 receptor is expressed the greatest in the lungs, it causes a respiratory illness as a result, and the virus is commonly found in nasopharynx area [36,41,42]. Once a virus binds to a cell, it penetrates the membrane, allowing for entry into the cell. Uncoating then occurs and the genome is released into the cytoplasm of the cell. The synthesis of viral proteins and genome can then occur. Viral proteins and genome are packaged into virions and they are matured before being released from the cell-by-cell lysis, budding, or exocytosis to cause further infections.

Upon viral infection, infected cells and viral particles are often taken up by antigen-presenting cells, which present viral antigens to T-cells to trigger an immune response. This immune response triggers the formation of antibodies against the virus to prevent it from spreading. The produced antibodies are often specific for viral surface proteins, such as HA, NA, spike proteins, and envelope proteins [36,40,43,44,45,46]. The binding of antibodies to these proteins can neutralize the virus, thus preventing its entry into a cell. When considering these biological reactions, most vaccines try to stimulate the production of antibodies against viral surface proteins to neutralize them and prevent their entry [40,43,45].

Biosensors can be designed to detect the virus or the antibodies produced against a virus. Viral surface antigens are the easiest to detect, as they are displayed on the outer surface of the virus. Accordingly, the biorecognition element of these biosensors can carry the receptors or the antibodies for these antigens, allowing for strong attachment to the biosensor [26,47,48]. Conversely, biosensors can be developed using a virus antigen for detection of virus induced antibodies. Using these antigens would be most beneficial because antibodies are the most produced against surface proteins. Finally, biosensors can be designed to detect the viral genome. This is done by immobilizing a complementary probe to the viral genome, or its genome transcription product, to the biorecognition surface of the biosensor [49,50]. Hybridization with the viral genome indicates its presence. While this method allows for higher specificity, it comes with the caveat of requiring additional sample preparation steps. 

## 3. Electrochemical Biosensors

Different electrochemical techniques can be used as transduction element for developing electrochemical biosensors, in which the biochemical reactions are transduced into electrical signals that are then detected through amperometric, potentiometric or impedimetric means. Electrochemical biosensors are advantageous as they require simple instrumentation, are highly sensitive, cost-effective, and they have the capability of miniaturization [12,13,47]. Electrochemical biosensors usually use the three-electrode cell configuration; this consists of a working, counter, and reference electrode. The working electrode is usually modified with viral proteins, a probe complementary to viral genome, or antibodies that are specific to that virus to allow detection of viral antibodies, genome, or proteins respectively [12,51]. Further modification using carbon or metallic nanostructures improves sensitivity by enhancing immobilization of recognition elements, or binding of target molecules due to increasing the surface area [13]. Katz et al. [48] summarized the metallic and semiconductive properties that were achieved from carbon nanotubes (CNT) and their potential integration with biomaterials for the modification of biosensor devices. Electrochemical readout is provided via voltammetry techniques, such as amperometry, cyclic voltammetry (CV), differential pulse voltammetry (DPV), and square-wave voltammetry (SWV), while using potentiometric apparatus as well as impedimetric means using electrochemical impedance spectroscopy (EIS) [13]. 

Amperometric methods measure the current occurring due to a redox reaction of an electroactive species in response to the applied electrical potential to the working electrode [52]. The measured current is indicative of electron transfer within the sample and at the electrode surface and, thus, the concentration of the analyte. CV is a linear-sweep method, in which the electric potential is swept in cycles and the corresponding current is measured. On the other hand, DPV and SWV use pulse voltammetry, in which the electric potential pulses are applied at periodic intervals, providing the advantage of improved speed and sensitivity [16,52,53]. Field-effect transistor (FET) based biosensors are another method used for the detection of pathogens [54,55,56,57,58,59]. Similar to three-electrode cells, FET consists of three terminals: source, gate, and drain. Bioreceptors are immobilized on to the gate that is connected to the source and drain electrodes. A potential is applied to the gate upon recognition of an analyte. This leads to a change in conductivity being sent through the source-drain channel, based on which molecules are detected. FET provides an increased sensitivity and easier fabrication as compared to their predecessor bipolar junction transistors [55]. Hence, FET detect pathogens through changes in conductivity between source-drain channels that arise from the electric field of the sample environment. Potentiometric methods measure electrical potential in response to the applied current [52,60]. On the other hand, EIS operates by measuring resistance (impedance) changes, which occur at the electrode-electrolyte interface due to molecular capturing. The EIS technique can be used to measure charge transfer resistance (Rct), solution resistance, and double layer capacitance. These parameters show the electrochemical behavior of the working electrode. The EIS biosensors are label-free techniques that determine the analyte by monitoring the changes in Rct while using an external redox probe, such as ferri/ferrocyanide redox couple. Excellent review articles discussed the principle and application of the EIS [61,62,63]. 

### 3.1. Influenza Biosensors

Yearly vaccines are currently the most effective way to protect against influenza [64]. These vaccines consist of inactivated external coat proteins, specifically hemagglutinin (HA) and neuraminidase (NA), which trigger an adaptive immune response. HA is an antigenic glycoprotein that is required for the entry of influenza into the cell. It has a high affinity for specific sialic acid glycans present on plasma membranes, allowing for attachment and entry by endocytosis [65]. NA is an enzyme that cleaves sialic acid residues, allowing the budding of the virus from the infected cell. Effective vaccines are those that induce specific antibody formation against HA and NA when administered [64]. Because HA and NA have multiple subtypes, it is often difficult to predict which subtype combination will affect the population. Therefore, the successful detection of HA, NA, and antibodies against HA and NA can allow for greater efficacy in vaccine development. These are currently detected using ELISA; however, a variety of electrochemical biosensors have been designed with this task in mind. Additionally, electrochemical biosensors utilizing the same principle of detecting HA, NA, or antibodies specific to HA and NA have been designed to use as diagnosis tools. The advantages of using biosensors over ELISA allow for smaller sample requirements and lower cost [16].

Recently, organic electrochemical transistors (OECT) have gained attraction for multiple immunosensor designs, due to their biocompatibility and stability [66,67,68]. OECTs are transistors that are composed of an organic semiconductor film between two metal electrodes immersed in an electrolyte solution. The organic film acts as a channel in which ions from the electrolyte solution can move into and out of it; this causes changes in doping, which lead to changes in conductivity. [69]. Polymers of 3,4-ethylenedioxythiophenepoly (EDOT) are often used to design the films, due to its conductivity, stability, and biocompatibility [70,71]. Hai et al. [67] reported a label-free OECT for the potentiometric detection of avian and human influenza A virus. The developed OECT was constructed by doping poly(EDOT) with poly(styrenesulfonate) (PEDOT:PSS) on a glassy carbon electrode. PEDOT was further covalently co-polymerized with oxylamine containing EDOT derivative. Oxylamine reacts with the reducing end of sialic acid containing trisaccharide 2,6-sialyllactose or 2,3-sialyllactose, to specifically bind to human and avian influenza, respectively, via the HA protein [67]. Because the influenza virus particle carries a net negative charge at pH 7.4, its binding to the trisaccharide increases the carrying density of the polymer, which allows the detection of the virus by potentiometry using an electrometer. A limit of detection (LOD) of 0.013 hemagglutinating unit (HAU) was achieved, providing promising use for future diagnosis. Multiple other biosensors have also focused on the high affinity of HA to bind to sialic acid glycans [66,72]. Krejcova et al. [72] targeted this property by modifying CdTe quantum dots that were conjugated to sialic acid glycans for the specific detection of HA. The glycan-conjugated magnetic nanoparticles were used as a biorecognition element to detect HA in vaccine samples using differential pulse anodic stripping voltammetry (DPASV) and DPV. A LOD of 62.5 pmol/L was achieved, which, according to the authors, is comparable to other nanoparticle-based or electrochemiluminescence immunosensors. Similar magnetic nanostructure designs have been used to fabricate other immunosensors for influenza detection [56,73]. While the HA-glycan interaction remains prominent in influenza immunosensor design, NA, the other crucial antigenic surface protein, has also been targeted by several techniques [74,75].

Other nanostructures, such as carbon nanostructures, have attracted appreciable attention as well for influenza detection, being employed in multiple electrochemical designs [69,74,76,77]. Using nanostructures greatly improves the sensitivity of detection, due to their small size and large surface area to volume ratio of nanoparticles [78]. Among the nanostructures, reduced graphene oxide (rGO) has been the most appealing, due to its large surface area, high charge carrier mobility, and high electrical conductivity [69,77]. Joshi et al. [77] introduced a label-free electrochemical immunosensor using thermodynamically decomposed rGO (TrGO) to detect the influenza A virus subtype H1N1. The TrGO film that was obtained from shellac was cast onto indium tin oxide/glass electrodes through 1-pyrenebutanoic acid succinimidyl ester (PBSE) as a linker to provide the recognition surface. Using EIS for measurement, they achieved a detection limit of 33 plaque forming units (PFU)/mL in diluted saliva, which, according to the authors, is suitable for most clinical detections of influenza in saliva and comparable to similar immunosensors [77].

**Figure 2:**
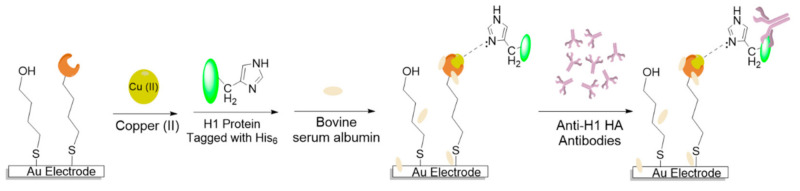
Schematic representation of electrochemical immunosensor for detection of anti-hemagglutinin (HA) antibodies. Au electrode modification with a mixture of 4-Mercaptobutanol (MBT) and dipyrromethene (DMP) followed by addition of Cu (II), His6-H1 HA tag, and Bovine serum albumin (BSA). The resulting His6-H1 HA/DMP/Cu (II) complex is able to detect anti-HA antibodies. Adapted from Mikuła et al. [79] under Creative Commons Attribution License.

Similarly, other immunosensors have been developed for detecting anti-HA antibodies [73,79,80,81]. Anti-HA antibodies appear in high concentrations in the blood during an influenza infection to neutralize the virus before cellular entry [65]. Mikuła et al. [79] designed an electrochemical biosensor by modifying the gold electrode with a mixed solution of 1 mM 4-Mercaptobutanol (MBT) and 0.01 mM dipyrromethene (DPM). DPM was bonded to Cu (II) to form the DPM-Cu (II) complex on the gold electrode. The DPM-Cu (II) complex allowed for the coordinate bond formation of histidine tag H1 subtype of HA (His6-H1 HA) with Cu (II), which acted as the biorecognition element to detect the anti-H1 HA (Figure 2). The authors used SWV to detect antibody binding. SWV uses waveforms that are comprised of large-amplitude symmetrical square waves superimposed on a base staircase waveform [82]. The net current obtained at high scan rates show high sensitivity and discrimination of the background current, which allows for the quantification of the difference between redox peaks while eliminating the capacitive current [82]. Mikuła et al. [79] were able to detect the antibodies in mouse sera that was diluted 10^9^-fold. This displayed better sensitivity than ELISA by a magnitude of 10^7^ [83]. Jarocka et al. [80] also used this technique to detect anti-H5 antibodies. They tested vaccinated hen sera for the presence of anti-H5 antibodies and managed to detect antibodies that were diluted to 7 × 10^6^ and 7 × 10^4^ for high and low responders, respectively. This was lower than the concentration limits of ELISA, providing promising use as a fast-immune surveillance technique.

### 3.2. Human Immunodeficiency Virus Biosensors

Human immunodeficiency virus (HIV) is known to be incurable in most instances [84]. One of the strategies for controlling HIV incidence often involves testing for HIV as early as possible to start treatment, such as anti-retroviral treatment (ART). This strategy lowers the viral load within the patients, reducing the chance of spreading the virus. This treatment requires lifelong monitoring of the viral load and it is typically done through Nucleic Acid testing (NAT)-based viral load test or CD4+ cell counting through flow cytometry [10]. However, these tools are complex, expensive, and require adequate laboratory infrastructure. This creates a need for an alternative assay that is cheaper and portable. Additionally, HIV is known to have a long latency period; hence, carriers may be asymptomatic for months to years before symptoms appear and possible progression to acquired immunodeficiency virus (AIDS) occurs [84]. During this time, the viral load is very low; hence, a very sensitive diagnostic test is needed for detecting HIV at this stage. HIV is generally examined using ELISA, which detects anti-HIV antibodies (IgG or IgM), but this method calls for a window period after infection to allow antibodies to form, where this period can take up to several weeks of post-infection in some cases [85]. Currently, other methods for the fast detection of HIV specific proteins, or genes are being explored. Electrochemical biosensors have been designed given this errand, being frequently intended for specific and sensitive recognition of HIV proteins or genome.

**Figure 3:**
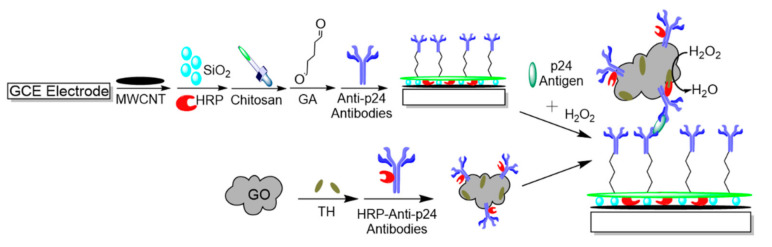
Schematic procedure of glassy carbon electrode (GCE) modification to detect p24 antigen. GCE modified with MWCNT/SiO2/HRP matrix, followed by the addition of chitosan and glutaraldehyde (GA) for attachment of anti-p24 antibodies. Graphene oxide (GO) modified with HRP-anti-p24 antibodies and thionine (TH) are added along with human immunodeficiency virus (HIV) p24 antigen and hydrogen peroxide for the detection of TH oxidized products. Adapted from Fang et al. [87] with permission from Elsevier.

HIV protein 24 (p24), which is a component of the HIV capsid, is often the target molecule for detection by many biosensors [86,87,88,89,90]. However, the low sensitivity of detection of many current biosensors is the main limitation for using p24 as a target. Electrochemical immunosensors have overcome this limitation by fabricating biosensors using nanomaterials to increase sensitivity [86,87,88,89]. Fang et al. [87] fabricated an electrochemical biosensor that is based on a sandwich immunoreaction for the detection of p24 antigen. They modified a glassy carbon electrode (GCE) using multiwalled carbon nanotubes (MWCNT), followed by silica (SiO_2_) and horseradish peroxidase (HRP), creating a matrix for immobilization. The matrix was modified while using chitosan and glutaraldehyde (GA) to allow for the attachment of anti-p24 antibodies onto the MWCNT/SiO_2_HRP matrix. Separately, HRP-modified anti-p24 antibodies were linked to nanocarrier graphene oxide (GO) and thionine (TH), forming GO/TH/HRP-anti-p24 antibodies. In the sandwich immunoreaction, both of the antibodies on the MWCNT/ SiO_2_/HRP and GO/TH bind to the antigen, where the addition of hydrogen peroxide causes the amplification of the signal through the enzymatic oxidation of TH. Figure 3 shows a schematic of this fabrication. The amplified signal was measured using DPV that detected p24 antigen in the linear concentration range of 0.5–8500 pM, with a detection limit of 0.15 pM.

Cerrutti et al. [91] focused on detecting anti-HIV p17 antibodies while using a layer-by-layer (LBL) method to immobilize lignin with HIV p17 antigen (p17 is a matrix structural protein). The lignin/p17 peptide LBL films were produced through alternating immersion of a gold electrode into solutions of lignin and p17 antigen. Using EIS, the authors were able to detect the presence of antibodies from a concentration range of 0.1 nM to 100 nM. However, as previously mentioned, targeting HIV antibodies has the caveat that it cannot be used during the early stages of infection, as antibodies have not formed, hence limiting this biosensor from testing during early stages of infection. Instead, this biosensor is used during vaccine development to determine the effectiveness of a vaccine during clinical trials.

FET are also commonly used to detect HIV [90,92,93]. Fatin et al. [92] developed a novel FET based biosensor using MWCNT as a layer for the immobilization of RNA aptamer as a probe to detect HIV Tat protein. Tat antigen is a regulatory protein that increases the viral transcription efficiency of HIV double strand DNA (dsDNA), thus increasing the number of transcripts made. The binding of Tat protein to RNA aptamer was detected through changes in voltage that occurred due to a decrease in the current flow in the concomitant gate. The authors were able to achieve a detection limit of 600 pM. The authors reported on an interference from other HIV proteins, such as Nef and p24 with the Tat protein; however, their signals remained much less intense than the Tat signal. Majid et al. [93] designed a novel liquid ion gate FET for the detection of the HIV genome using a modified Nickel oxide (NiO) sensing layer. The NiO layer was deposited on a glass substrate using radio frequency reactive magnetron sputtering followed by modification using (3-Aminopropyl)-triethoxysilane and GA. A 5-amino-modified single strand DNA (ssDNA) probe (5′NH2-GGG GGG CCA AGG CCC AGC CCT CAC ACA-3′) was then covalently immobilized to the NiO sensing layer via GA. A change in voltage was detected by FET upon hybridization with the complementary HIV genome, giving a detection limit of 0.3 aM. This method was also able to differentiate between two base mismatch sequences, thus exhibiting good selectivity [93].

Other HIV electrochemical biosensors have been developed to also detect the HIV genome [94,95,96,97,98,99,100,101]. Gong et al. [95] developed an impedimetric biosensor (ssDNA/graphene-Nafion/GCE) for the detection of the HIV gene. They modified a GCE electrode while using a graphene-Nafion composite solution, followed by treatment with a ssDNA as a capture probe, which was absorbed on to the composite via π–π* stacking interactions. Upon hybridization with the complementary HIV gene, the dsDNA is removed from the electrode, causing a decrease in Rct. The authors were able to achieve a detection limit of 23 fM. Shamsipur et al. [102] also reported on a biosensor using a sandwich nanocomposite film to detect the HIV gene. The sandwich nanocomposite was prepared through electro-polymerization of p-aminobenzoic acid (PABA) onto rGO modified GCE. This was followed by the electrodeposition of AuNPs, that results in the formation of the AuNPs/PABA/rGO/GCE platform. Subsequently, the ssDNA (5′-SH-GCT TGC CAA TGA TCT GTC CA-3′) was immobilized on to the AuNP layer through a thiol bond. A LOD of 37 aM was obtained while using EIS to detect the hybridization with the HIV gene. The authors also reported a decreased in the signal intensity with an increase in mismatch bases indicating the selectivity of the electrode.

### 3.3. Ebola Virus Biosensors

Ebola virus (EBV) has a high case fatality rate, ranging between 44–90% [103]. It is highly transmissible by direct contact with bodily fluids from the living or dead infected individuals and it even has a low chance of transmission through inanimate objects contaminated with infected bodily fluids [103]. EBV is typically diagnosed using real-time reverse transcriptase-polymerase chain reaction (RT-PCR) [104]. RT-PCR usually detects EBV infection in patients after 3–6 days of symptoms onset, and often giving false negatives if tested too early. Hence, this method is not reliable for detection during the early stages of infection and it often needs repetition if negative results come up. Additionally, most current outbreaks have occurred in resource-poor area, such as African villages, with a lack of adequate infrastructure. Therefore, RT-PCR, which requires trained personnel and expensive equipment, cannot be used. EBV detection is further complicated, as IgG and IgM antibodies do not develop in all cases; thus, using serology to detect EBV is only applicable to a subset of patients [104]. The need for an early diagnosis method of EBV is critically important because once symptoms begin, progression to Ebola Hemorrhagic fever is swift. Electrochemical biosensors stand out as a diagnostic method due to their low-cost, rapid response, and high sensitivity.

**Figure 4:**
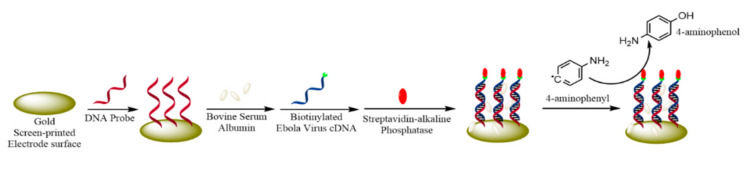
SPE is modified using a DNA probe complementary to Ebola virus (EBV) cDNA. Biotinylated EBV cDNA is added, which binds to streptavidin-alkaline phosphatase allowing for the recognition of hybridization through the conversion of 4-aminophenyl to 4-aminophenol. Adapted from Ilkhani et al. [106] with permission from Elsevier.

Screen printed electrodes (SPEs) have been regarded for their user-friendliness, low cost, low sample and waste size, and their ability to miniaturize setup [105]. This makes them environmentally friendly and very attractive to use for designing electrochemical biosensors. These are composed of working, counter, and reference electrodes that are printed on a substrate for detection. SPE has often been employed to design the DNA-based electrochemical biosensors [105]. These biosensors contain a capture single-stranded DNA (ssDNA) as the biorecognition element immobilized on the electrode surface, which is complementary to the target DNA. Once the target molecule is added, hybridization occurs, which can be measured through a redox label that is placed on either component, or through the addition of a redox label that reacts with the hybridized product. Ilkhani et al. [106] designed an electrochemical biosensor using a gold SPE and a DNA capture probe complementary to synthetic EBV complementary DNA (cDNA). Figure 4 shows a schematic representation of the fabrication process. The thiolated ssDNA capture probe (5′-TTG TAC GAA GCT GTA CAT AAA TT-(CH2)-SH) was immobilized on the gold surface via a thiol bond. Bovine serum albumin (BSA) was then added to fill and deactivate any free spaces in the electrode in order to minimize the non-specific adsorption. Subsequently, a biotinylated EBV cDNA strand, which was complementary to the ssDNA probe, was added, allowing for hybridization to occur. Subsequently, streptavidin-alkaline phosphatase was introduced, which bonded to the hybridized DNA via a biotin-streptavidin conjugation bond. Afterwards, the streptavidin-alkaline phosphatase was enzymatically reacted with 4-aminophenyl phosphate solution to produce 4-aminophenol. EIS and DPV were used to determine the hybridization and 4-aminophenol formation, respectively. This biosensor was able to detect EBV linearly in the tested concentration range of 10–75 nM, with a limit of detection (LOD) of 4.7 nM. However, the authors did not test a real EBV sample.

**Figure 5:**
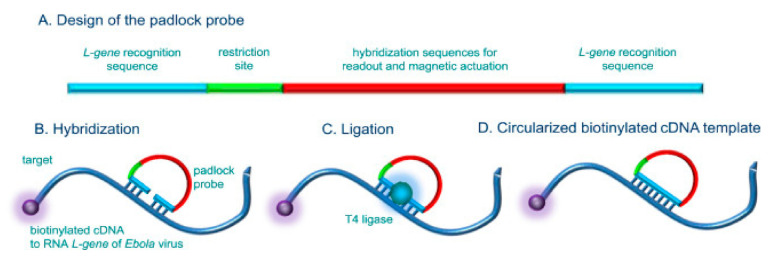
Diagram of padlock probe and its circularization upon hybridizing. (**A**) Padlock probe composition containing two target-complementary sequences (L-gene recognition sequence) connected by a linker sequence containing restriction and readout sites. (**B**–**D**) Hybridization of probe with biotinylated cDNA (mimicking viral EBV L-gene) allowing for ligation using T4 ligase and subsequent circularization. [107] Reused with permission from Elsevier.

**Figure 6:**
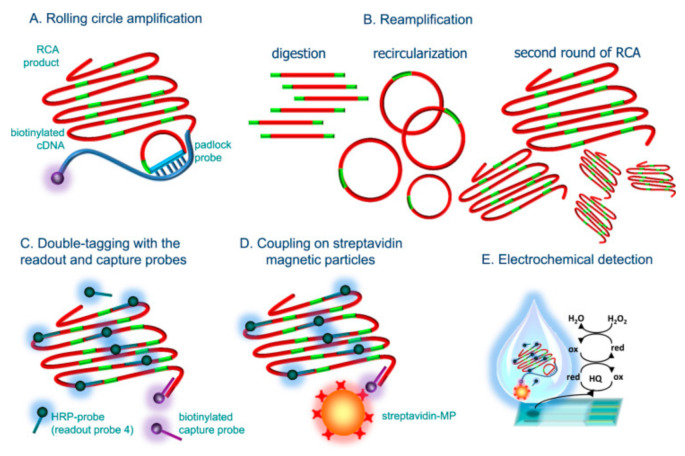
Schematic representation of electrochemical detection following hybridization of probe with target sequence. (**A**) RCA of circularized probe occurs followed by (**B**) its digestion and subsequent recircularization allowing for a second round of RCA to occur. (**C**) DNA products were double tagged using HRP probe and biotinylated capture probe, (**D**) which allowed for coupling of biotinylated DNA onto streptavidin magnetic particles. (**E**) Finally, enzymatic reaction of HRP using H2O2 as a substrate was detected. [107] Reused with permission from Elsevier.

Carinelli et al. [107] designed a rapid EBV test using an amplification step before electrochemical detection. They used rolling circle amplification (RCA) to amplify the EBV genome before detecting while using SWV. RCA is an amplification method for circular DNA that follows similar principles as PCR. The main difference being that RCA does not require temperature cycling and, instead, can perform continuing replication of circular DNA while using φ29 DNA polymerase [108]. Being initiated by a primer, the continuous synthesis of long linear concatenated replicas occurs until termination. Carinelli et al. [107] designed padlock probes, which were linear oligonucleotides that contained two target-complementary sequences connected by a target-independent sequence. The complementary sequence to EBV L-gene (L-gene found in most EBV) served as the target-complementary sequences. Figure 5 shows diagrammatic representation. Magnetic particles that were covered in streptavidin were attached to biotinylated EBV cDNA via a biotin-streptavidin bond. The padlock probe was able to hybridize with the L-gene sequence on the cDNA and, once hybridized, the probe was circularized by enzymatic ligation, connecting the two target-complementary sequences. Once circularized, RCA using φ29 DNA polymerase could occur. The product was then digested using restriction enzymes and reamplified through a second round of RCA. Repetitive sequences in the amplified DNA were labelled using HRP that was conjugated to a complementary probe and a biotinylated capture probe. The capture probe allowed for the coupling of the amplified DNA to streptavidin magnetic particles on the SPE. The enzymatic product of HRP was detected using the SWV technique. Figure 6 shows a summary of the procedure. The authors were able to achieve a LOD of 33 cDNA molecules. Although multiple amplification steps are required before detection, which makes this method very complex, the authors indicated that the total assay time took less than 2.5 h, thus providing a fast and sensitive detection method.

FET has also been utilized to design electrochemical biosensors for EBV detection, due to their low cost and ease of use [58,59]. Jin et al. [59] modified a FET using reduced graphene oxide (RGO) in order to build a biosensor to detect inactivated EBV. Anti-EBV glycoprotein antibodies were immobilization onto RGO modified FET through PASE, a crosslinker molecule. Once modified, EBV in serum was incubated to allow detection, which was measured through a shift in Dirac voltage. The Dirac voltage is the peak voltage that is seen in the V-shaped curve of graphene FET due to its ambipolar properties. The authors were able to obtain a LOD as low as 2.4 pM.

### 3.4. Zika Virus Biosensors

Zika virus (ZIKV) is known to be difficult to diagnose. since most infected patients are asymptomatic or show mild symptoms reminiscent of other flavivirus infections, such as chikungunya and dengue [109], which is often misdiagnosed with these infections [110]. Furthermore, ZIKV is linked to microcephaly in newborns with infected mothers and Guillain–Barré syndrome, which means that early detection is critical [109,110,111]. Currently, the most common diagnostic method is RT-PCR, which can detect ZIKV genome in biofluid samples, such as saliva, human serum, or urine [112,113,114,115]. However, this method requires trained personnel and expensive equipment, and it is time-consuming to perform, as mentioned before. Hence, it may not be accessible in poor regions, where the highest number of ZIKV cases are seen. ELISA has also been used for ZIKV diagnosis [116]. It is specifically used to detect anti-ZIKV antibodies in serum; however, cross-reactivity with antibodies similar to ZIKV antibodies (such as dengue virus) makes this method unselective against ZIKV. Therefore, there is a need for a diagnostic tool that is simple, cost-effective, selective, and sensitive for ZIKV, to allow for detection, even at early stages of the infection. Electrochemical biosensors have all of the above features to overcome the challenges in ZIKV diagnosis [117].

**Figure 7:**
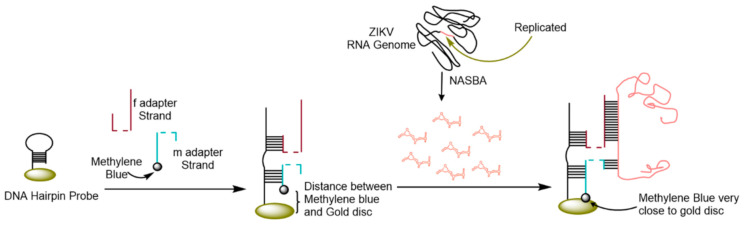
Fabrication of a four-way junction (4WJ) electrode using a gold disc electrode, a DNA hairpin probe, and two adaptor strands complementary to the hairpin probe. A specific section of ZIKV RNA genome is replicated by NASBA and it is also complementary to the adaptor strands. The binding of adaptor sequence and ZIKV RNA brings methylene blue redox label closer to gold disc electrode, increasing the detected current. Adapted from Lynch et al. [118] with permission from Elsevier.

Genome-based electrochemical biosensors have been among the most popular technique to detect ZIKV; this is due to their sensitivity and ability to distinguish ZIKV from dengue and other similar viruses, allowing for detection at early stages of infection [50,118,119,120]. Genome-based electrochemical biosensors often employ nucleic acid sequence-based amplification (NASBA) before detection in order to amplify low concentrations of RNA in sample [118,119]. NASBA follows a similar process as PCR for the amplification of RNA sequences. Using thermal denaturation, stable RNA polymerase, reverse transcriptase, and Ribonuclease H, it greatly amplifies the concentration of the target RNA in a sample [121]. Lynch et al. [118] used the NASBA technique along with a four-way junction (4WJ) design to selectively detect ZIKV RNA. The 4WJ was built using a universal DNA hairpin probe that was immobilized on a gold disc electrode via a thiol bond, and then incubated with two DNA adaptor strands, m and f. The m adaptor sequence allowed for high selectivity of the ZIKV RNA, while the f adaptor sequence helped in unwinding of the secondary structure of the genome. The m adaptor strand also contained a methylene blue redox label that is brought close to the electrode surface upon complementary binding to the ZIKV RNA, which increases the charge on the disc, and the current as a result. The two adaptor strands are complementary to the DNA hairpin probe and to a fragment of ZIKV RNA genome. Figure 7 shows the stepwise fabrication of this biosensor. The increase in current resulting from the binding of ZIKV RNA allows for detection by square-wave voltammetry. The authors were able to achieve a detection limit of approximately 0.3 fM and they were able to discriminate the ZIKV RNA against dengue and other similar flaviviruses (West Nile virus). Mills et al. [119] used the same 4WJ method to detect for ZIKV and dengue virus separately. Using SWV, they were able to obtain an LOD of 0.98 nM of ZIKV RNA. Additionally, they showed that the biosensor could be regenerated and reused by rinsing with urea and water.

Other electrochemical biosensors have implemented the use of ZIKV antibodies for virus detection [50,121,122,123,124]. However, differentiation between ZIKV and Dengue virus is challenging, since the antibodies that are produced by ZIKV are often cross-reactive with Dengue viruses [123]. Cabral-Miranda et al. [122] developed a novel immunosensor that contained recombinant ZIKV non-structural protein 1 (NS1) and recombinant domain III of envelope protein (EDIII) as two antigens for the detection of anti-NS1 and anti-EDIII antibodies, respectively. NS1 is a replicase component that is secreted into the extracellular matrix along with the ZIKV virion, while the envelope protein is the major glycoprotein in the viral envelope [124,125]. EDIII, in particular, is involved with receptor binding and fusion with the host cell, hence antibodies against it prevent the virus from entering into the cell [124]. These two proteins are initially connected during replication but are later cleaved upon maturity. Cabral-Miranda et al. [122] used a suspension of carboxylated carbon nanotubes, which were reacted with phenylenediamine to allow for binding to EDIII and NS1. This modification was applied on carbon SPEs to allow immobilization of EDIII and NS1, and antibodies against them were added. Using EIS and SWV, the authors were able to detect anti-NS1 antibodies and anti-EDIII antibodies with an LOD of 17 fM and 53 fM, respectively. Additionally, they were able to differentiate ZIKV antibodies from dengue protein antibodies, and they demonstrated that their biosensor was more sensitive (able to detect a lower concentration of both anti-NS1 and anti-EDIII antibodies) than traditional ELISA techniques. A few other studies have also focused on NS1 and the envelope protein [126,127,128]. Although NS1 is commonly found in many flaviviruses, the ZIKV NS1 protein has been shown to have a unique conformation and characteristics that differentiate it from other flaviviruses’ NS1 proteins. Hence, it can be used to differentiate between ZIKV and other flaviviruses, such as dengue. Faria et al. [127] targeted this property and developed a biosensor using ZnO nanostructures and anti-NS1 antibodies for the detection of NS1. They grew ZnO nanostructures on a printed circuit board using chemical bath deposition. Anti-ZIKV NS1 antibodies were then immobilized on to the ZnO nanostructures via cystamine and GA. While using CV, they tested for antigen binding in urine and found the biosensor to be very sensitive with a LOD lower than 1 pM. Additionally, they were able to differentiate the response from dengue NS1 providing a selective biosensor for ZIKV NS1 detection. Tancharoen et al. [129] focused on the whole ZIKV instead of individual proteins. Their biosensor was based on surface imprinted polymers and GO composites. Using this platform, they were able to detect ZIKV using CV and EIS, and they achieved a detection limit that was close to the LOD of RT-PCR [129].

Despite the significant strides in ZIKV detection, there still remains a need for a system that is portable and can easily be mass produced at a low cost [130]. Additionally, while electrochemical biosensors have been successful in ZIKV detection, there is still a need to test on clinical samples in order to find its clinical relevance. This is especially important for detection during early stages of infection, as a very low viral titre is present.

### 3.5. Severe Acute Respiratory Syndrome Coronavirus 2 Biosensors

SARS-CoV-2, which is the causative agent of the current COVID-19 pandemic, has currently affected more than 20 million people worldwide, but the number is predicted to be much higher as many people go untested. Because no specific drug or vaccine currently exists to treat COVID-19, the best method to control the COVID-19 cases is through early diagnosis and management. This has best been highlighted in South Korea and Germany, where high levels of testing allowed for them to curb the rate of infections [131,132]. This has brought a demand for point-of-care diagnostics that can be deployed in secondary care settings, airports, and even in patients’ homes, allowing for increased accessibility to viral testing. So far, many tests rely on nucleic acid diagnostics with the most common one being real time RT-PCR, which has been employed globally to deal with the pandemic [133,134]. The current RT-PCR technique detects envelope (E) protein and RNA-dependent RNA polymerase genes, as these are highly specific for SARS-CoV-2 RNA. This technique is very selective with high sensitivity that is able to differentiate the SARS-CoV-2 from other coronaviruses [135]. However, as previously mentioned, this method is a time-consuming process requiring trained personnel and adequate laboratory infrastructure, which prevents it from being employed in remote locations or poorer countries. Hence, an effective point-of-care diagnostic tool is needed, and electrochemical biosensors have been considered to fill this void. We also need to consider portability and economic aspects of the biosensor to build an effective point-of-care diagnostic [136,137]. For portability, this includes the need for devices to be miniaturized, so they are easily transported and can be easily stored. Additionally, they need to be robust and require little or common detection apparatus, so they can be used in remote and poor regions. For economic aspects, the biosensor needs to be of low cost and feasible for mass production in a short time to allow mass-testing. SPE and FET methods can be used in order to construct low cost and effective biosensors to test SARS-CoV-2 proteins, such as spike protein or SARS-CoV-2 genome.

Tripathy et al. [138] previously developed a miniaturized electrochemical biosensor that was based on DNA hybridization to detect dengue and other viral or bacterial infections. They revised and tailored this biosensor to propose the detection of SARS-CoV-2. They modified a titanium working electrode by the electrodeposition of AuNP. This is followed by immobilization of a ssDNA probe, which is complementary to SARS-CoV-2 RNA or its corresponding c-DNA, via a gold-thiol bond. The probe needs to be complementary to a specific SARS-CoV-2 sequence to allow for high selectivity on detection. Based on previous RT-PCR techniques, a few genes have been found to be specific to SARS-CoV-2; E protein, RNA-dependent RNA polymerase, and helicase genes, where the latter two did not cross-react with other human coronaviruses or other respiratory viruses [139]. However, the drawback to this method is the requirement of sample extraction and preparation before testing. This adds additional steps before diagnosing can occur, which has the possibility of affecting diagnostic accuracy and increases the time required for analysis [133]. Hence, a diagnostic method that can directly detect SARS-CoV-2 is essential for rapid and accurate diagnosis. Detecting SARS-CoV-2 surface proteins, such as the spike protein, can allow for diagnosis without the need for specific sample preparation, as compared to the previously mentioned nucleic acid diagnosis (RT-PCR), which requires RNA extraction and amplification.

**Figure 8:**
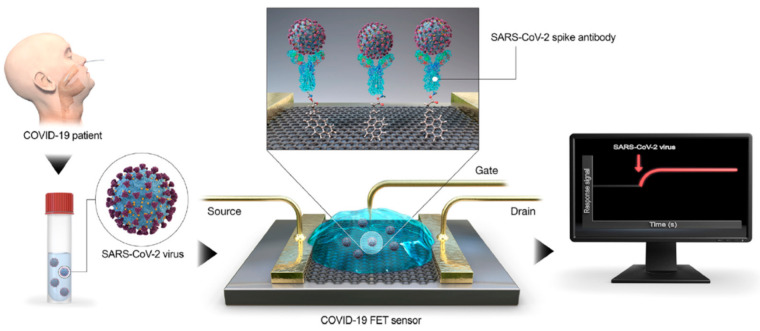
Field-effect transistor (FET) biosensor utilizing spike protein antibodies for detection of re acute respiratory syndrome coronavirus-2 (SARS-CoV-2) obtained through nasopharyngeal swab samples and stored in universal transport medium. This process requires no sample preparation [140]. Reused with permission from ACS publishing.

Seo et al. [140] designed a FET based biosensing device for detecting SARS-CoV-2 using spike protein antibodies as the detection probe. The FET was coated on graphene sheets, followed by the immobilization of anti-spike protein antibodies (SpAb) through coupling agent 1-pyrenebutyric acid N-hydroxysuccinimide ester (PBASE), which acted as a probe linker. They tested the performance of the FET using the antigen protein, cultured virus, and nasopharyngeal swab samples from COVID-19 patients. The FET biosensor was able to detect the spike protein antigen in both phosphate-buffered saline and universal transport medium (used to suspend nasopharyngeal swabs in clinical diagnosis) at 1 fM and 100 fM, respectively. The cultured virus and nasopharyngeal swab samples showed a LOD of 16 pfu/mL and 2.42 × 102 copies/mL, respectively. Mahari et al. [57] used a similar a technique to detect SARS-CoV-2. They developed a novel in-house built biosensor, eCovSens (National Institute of animal biotechnology, Hyderabad, India), while using a fluorine doped tin oxide (FDTO) electrode and AuNPs for the detection of SAR-CoV-2 spike protein. First, they immobilized anti-spike protein antibodies (SpAb) on the AuNPs through physisorption and electrostatic interactions to form AuNPs/SpAb. The device was prepared by coating a glass electrode with FDTO, followed by drop casting of AuNPs/SpAb. Figure 8 shows a summary of this fabrication. The binding of SARS-CoV-2 spike protein to SpAb was detected using DPV. Under optimum conditions, a detection of 1 fM to 1 μM was obtained, while, under spiked saliva samples, the authors were able to reach a LOD of 90 fM. Additionally, the biosensors were able to detect the spike protein within 10–30 s, thus providing a rapid detection and promising clinical use.

Serology testing is another method that has been employed to diagnose SARS-CoV-2 infection, with ELISA being the most common for detecting SARS-CoV-2 antibodies [141]. While no biosensor has been currently developed to detect for SARS-CoV-2 antibodies, this is another possible biosensor design. It is based on detecting for IgM and IgG SARS-CoV-2 antibodies that are produced in patients during the second week of viral infection [142]. Because antibodies take up to two weeks to form and, in some patients, there is no antibody formation, biosensors that are built on this principle are not effective for early diagnosis and as a screening tool. Instead, they can be used during vaccine development and trials to provide feedback on vaccine response. These biosensors would serve a crucial role in detecting for the formation of antibodies from the vaccines, as there is considerable attention towards developing high efficacy COVID-19 vaccines.

In summary, there is currently considerable attention into biosensors for early diagnosis of SARS-CoV-2 infection. It is possible to modify previously designed biosensors for the detection of COVID-19; however, while they have great clinical selectivity and specificity for the virus, they are still lower than the current gold standard, RT-PCR [137]. Although they may present future use as point-of-care diagnostics, further work still needs to be done in order to miniaturize and lower the cost of these biosensors. Currently, optical, electrochemical, and molecularly imprinted biosensors are being designed for the detection of COVID-19 [143,144,145,146,147,148,149], as seen in Table 1:

## 4. Conclusions

The COVID-19 pandemic has defined the global landscape since March 2020. As our current biggest challenge, COVID-19 has highlighted our vulnerability to viral infectious diseases, despite the advancements in medicine, hygiene, and healthcare. Moreover, much of our current knowledge in planning and controlling viral outbreaks stems from previous outbreaks, such as influenza (H1N1) pandemic, HIV global epidemic, Ebola epidemic, and Zika epidemic. However, COVID-19 has exceeded those in terms of overall mortality and socioeconomic impact. This highlights the tremendous importance of diagnostics as a tool to combat and control the level of spread. This review touched over the techniques and designs used to develop electrochemical immunosensors in the last five years for recent pandemics and epidemics (Table 1). The details of immobilization procedures, electrochemical response mechanism, and their applications have been also reviewed. During the past decades, extensive research has been performed into the sensitive and selective detection of viruses, where electrochemical techniques were often chosen over techniques, like PCR or ELISA, due to their rapid response, cost-effectiveness, simple fabrication, and requiring a small sample size. Electrochemical biosensing methodologies have achieved huge improvements for virus detection in terms of selectivity, sensitivity, specificity, and response time. Designing microfluidics and miniaturizing set-up provided low-cost fabrication and low waste production for electrochemical biosensors, which make them a potential platform as a POC tool. Despite electrochemical biosensors demonstrating very promising features, there are many challenges before they can substitute current diagnostic techniques or be used as POC tools. For instance, the lifetime of electrochemical biosensors can vary significantly, as well as the immobilization of nanomaterials and biological elements is crucial in minimizing false positives or negatives results. While some designs do show high sensitivity and selectivity, most of these biosensors have not been validated with real samples. Overall, great effort is required to improve current biosensors to make them portable, reusable, and capable of distinguishing between viruses. Finally, new advances in nanomaterials, nanofabrication technologies, and biomimetic surfaces can all be further explored for developing biosensors that continue to push the boundaries of viral detection. This will improve the management of the COVID-19 pandemic by getting people into quarantine and preventing the spread of the virus, as well as helping us prepare for future pandemics by allowing for faster response times in developing diagnostic tools.

## Figures and Tables

**Table 1:** Summary of performance characteristics of developed biosensors for Influenza, HIV, Ebola, Zika, and SARS-CoV-2.

Virus	Target	Recognition Element	Detection Method	LOD	Working Range	Ref
Influenza	H1N1 HA	2,6-sialyllactose /Oxylamine/(PEDOT:PSS)	FET	0.013 HAU	-	[67]
Influenza	H1N1	2,6-sialyllactose /Oxylamine/(PEDOT:PSS)	FET	0.025 HAU	-	[68]
Influenza	H1N1	Antibody/RGO/Cystamine modified gold electrode	Chronoamperometry and CV	0.5 PFU/mL	1–10^4^ PFU/mL	[69]
Influenza	H1N1 HA	Sialic acid/CdTe quantum dots	DPASV	62.5 pmol/L	-	[72]
Influenza	H9N2 NA	Graphene–Au hybrid nanocomposite SPE	EIS	10^−8^ U/mL	10^−8^ to 10^−1^ U/mL	[74]
Influenza	H9N2 matrix Protein	Fetuin-AuNPs and Anti-Matrix 2 Antibodies/Magnetic NPs	CV and Chronoamperometry	16 HAU	8–128 HAU	[76]
Influenza	H1N1	TrGO/PBSE/InTiO	EIS	33 PFU/mL	-	[77]
Influenza	Anti-H1 HA antibodies	His_6_-H1 HA/DPM-Cu (II)	SWV	-	-	[79]
Influenza	Anti-H5 HA antibodies	His_6_-H5 HA/DPM-Cu (II)	SWV	2.4 pg/mL	4.0 to 100.0 pg/mL	[80]
Influenza	H1N1/H1N5 HA	SPE-GO/Methylene Blue/Chitosan-protein A/Antibody	DPV	8.3 pM	25−500 pM	[81]
HIV	p24 antigen	GO/CNT/Silica electrode	CV and DPV	0.083 pg/cm^3^	10^−4^ to 2 ng/cm^3^	[86]
HIV	p24 antigen	anti-p24 antibodies/GA/Chitosan and GO/TH/HRP-anti-p24 antibodies	DPV	0.15 pM	0.5 to 8500 pM	[87]
HIV	Anti-HIV p24 antibody	p24 antigen/Chitosan/GA/Single-walled CNT-SPE	DPV	2 pM	10 pM to 1 nM	[89]
HIV	p24 antigen	Antibody/amine-functionalized graphene-FET	FET	100 fM	100 fM–1 μM	[90]
HIV	Anti-HIV p17 antibody	LBL to immobilize Lignin and p17 on Au electrode	EIS	-	0.1 nM–100 nM	[91]
HIV	HIV Tat antigen	RNA Aptamer/MWCNT-FET	FET	600 pM	-	[92]
HIV	Viral DNA	ssDNA probe/NiO/Liquid ion gate-FET	FET	0.3 aM	10^−18^ to 10^−8^ M	[93]
HIV	Viral DNA	ssDNA probe/AuNPs/ polymer-encapsulated quantum dots	SWV	0.2 fM	0.5 fM to 500 pM	[94]
HIV	Viral DNA	ssDNA/Graphene-Nafion/GCE	EIS	23 fM	10^−13^ to 10^−10^ M	[95]
HIV	Viral Genome	ssDNA/rGO/GCE	EIS	0.3 pM	10^−12^ to 10^−9^ M	[96]
HIV	Viral DNA	exonuclease III/ssDNA probe/Au Nanocluster/GCE	DPV	3 × 10^−17^ M	10^−16^ to 10^−10^ M	[97]
HIV	HIV DNA	Sandwich nanocomposite AuNPs/PABA/rGO/GCE	EIS	37 aM	-	[102]
Ebola	Viral Glycoprotein	Antibody/AuNPs/rGO-FET	FET	1 ng/ml	-	[58]
Ebola	Viral Glycoprotein	Antibody/rGO-FET	FET	2.4 pM	2.4 pM to 12 μM	[59]
Ebola	Viral cDNA	cDNA probe/Au-SPE	EIS and DPV	4.7 nM	10 to 75 nM	[106]
Ebola	Viral cDNA	HRP conjugated probe and Streptavidin-magnetic NPs-SPE	SWV	33 cDNA copies	-	[107]
Zika	Viral RNA	ssDNA/Poly(3-amino-4-hydroxybenzoic acid)-modified pencil carbon graphite electrode	SWV	25.4 pM	84 pM to 1.41 nM	[50]
Zika	Viral RNA	4WJ structure-Gold Electrode	SWV	0.98 nM	1 to 75 nM	[119]
Zika	Viral RNA	4WJ structure-Gold Electrode	SWV	0.3 fM	-	[118]
Zika	Viral RNA	AuNPs/graphite carbon nitrite/Zr- metal-organic gel	CV	0.1 nM	0.3 nM to 3 μM	[120]
Zika	Anti-NS1 Antibodies	Recombinant NS1/Carbon-SPE	EIS and SWV	17 fM	-	[122]
	Anti-EDIII Antibodies	Recombinant EDIII/Carbon-SPE	EIS and SWV	53 fM	-	
Zika	NS1	Antibodies/Graphene-FET	Amperometry	450 pM	-	[126]
Zika	NS1	Antibody/Cystamine/GAZnO nanostructure-print circuit board	CV	<1 pM	-	[127]
Zika	Envelope protein	Antibody/dithiobis(succinimidyl propionate)/Au microelectrode	EIS	10 pM	10 pM to 1 nM	[128]
Zika	ZIKV	Surface Imprinted Polymer and GO composite	CV and EIS	2 × 10^−4^ PFU/mL	-	[129]
SARS-CoV-2	Spike Protein	SpAb/AuNPs/FDTO	DPV	90 fM	-	[57]
SARS-CoV-2	Spike Protein	SpAb-PBASE/Graphene-FET	FET	16 pfu/ml	-	[140]

## Data Availability

Not applicable.

## References

[1] Nicola M., Alsafi Z., Sohrabi C., Kerwan A., Al-Jabir A., Iosifidis C., Agha M., Agha R. (2020). The socio-economic implications of the coronavirus pandemic (COVID-19): A review. Int. J. Surg..

[2] WHO Data and Statistics. https://www.who.int/hiv/data/en/.

[3] Kelly-Cirino C.D., Nkengasong J., Kettler H., Tongio I., Gay-Andrieu F., Escadafal C., Piot P., Peeling R.W., Gadde R., Boehme C. (2019). Importance of diagnostics in epidemic and pandemic preparedness. BMJ Glob. Health.

[4] Perkins M.D., Dye C., Balasegaram M., Bréchot C., Mombouli J.-V., Røttingen J.-A., Tanner M., Boehme C. (2017). Diagnostic preparedness for infectious disease outbreaks. Lancet.

[5] WHO Standardization of Terminology of the Pandemic A(H1N1) 2009 Virus. https://www.who.int/influenza/gisrs_laboratory/terminology_ah1n1pdm09/en/.

[6] WHO Director-General’s Opening Remarks at the Media Briefing on COVID-19. https://www.who.int/dg/speeches/detail/who-director-general-s-opening-remarks-at-the-media-briefing-on-covid-19---11-march-2020.

[7] Bedford J., Enria D., Giesecke J., Heymann D.L., Ihekweazu C., Kobinger G., Lane H.C., Memish Z., Oh M., don Sall A.A. (2020). COVID-19: Towards controlling of a pandemic. Lancet.

[8] Bramhachari P.V., Mohana Sheela G., Prathyusha A.M.V.N., Madhavi M., Satish Kumar K., Reddy N.N.R., Berde C.P. (2020). Advanced immunotechnological methods for detection and diagnosis of viral infections: Current applications and future challenges. Dynamics of Immune Activation in Viral Diseases.

[9] Velusamy V., Arshak K., Korostynska O., Oliwa K., Adley C. (2010). An overview of foodborne pathogen detection: In the perspective of biosensors. Biotechnol. Adv..

[10] Lifson M.A., Ozen M.O., Inci F., Wang S., Inan H., Baday M., Henrich T.J., Demirci U. (2016). Advances in biosensing strategies for HIV-1 detection, diagnosis, and therapeutic monitoring. Adv. Drug Deliv. Rev..

[11] Bhalla N., Jolly P., Formisano N., Estrela P. (2016). Introduction to biosensors. Essays Biochem..

[12] Thévenot D.R., Toth K., Durst R.A., Wilson G.S. (2001). Electrochemical biosensors: Recommended definitions and classification. Anal. Lett..

[13] Cho I.-H., Kim D.H., Park S. (2020). Electrochemical biosensors: Perspective on functional nanomaterials for on-site analysis. Biomater. Res..

[14] Sawant S.N. (2017). Development of Biosensors from Biopolymer Composites. Biopolymer Composites in Electronics.

[15] Sanati A., Jalali M., Raeissi K., Karimzadeh F., Kharaziha M., Mahshid S.S., Mahshid S. (2019). A review on recent advancements in electrochemical biosensing using carbonaceous nanomaterials. Microchim. Acta.

[16] Hammond J.L., Formisano N., Estrela P., Carrara S., Tkac J. (2016). Electrochemical biosensors and nanobiosensors. Essays Biochem..

[17] Clark L.C., Lyons C. (1962). Electrode systems for continuous monitoring in cardiovascular surgery. Ann. N. Y. Acad. Sci..

[18] Gregg B.A., Heller A. (1990). Cross-linked redox gels containing glucose oxidase for amperometric biosensor applications. Anal. Chem..

[19] Degani Y., Heller A. (1987). Direct electrical communication between chemically modified enzymes and metal electrodes. I. Electron transfer from glucose oxidase to metal electrodes via electron relays, bound covalently to the enzyme. J. Phys. Chem..

[20] Sage A.T., Besant J.D., Lam B., Sargent E.H., Kelley S.O. (2014). Ultrasensitive electrochemical biomolecular detection using nanostructured microelectrodes. Acc. Chem. Res..

[21] Bin X., Sargent E.H., Kelley S.O. (2010). Nanostructuring of sensors determines the efficiency of biomolecular capture. Anal. Chem..

[22] Zhang H., She Z., Su H., Kerman K., Kraatz H.B. (2016). Effects of bipyramidal gold nanoparticles and gold nanorods on the detection of immunoglobulins. Analyst.

[23] Kerman K., Mahmoud K.A., Kraatz H.-B., Kraatz H.B. (2007). An electrochemical approach for the detection of HIV-1 protease. Chem. Commun..

[24] Soleymani L., Fang Z., Sargent E.H., Kelley S.O. (2009). Programming the detection limits of biosensors through controlled nanostructuring. Nat. Nanotechnol..

[25] Kerman K., Saito M., Tamiya E., Yamamura S., Takamura Y. (2008). Nanomaterial-based electrochemical biosensors for medical applications. TrAC Trends Anal. Chem..

[26] Pingarrón J.M., Yáñez-Sedeño P., González-Cortés A. (2008). Gold nanoparticle-based electrochemical biosensors. Electrochim. Acta.

[27] Wang J., Liu A.G., Merkoçi A. (2003). Electrochemical coding technology for simultaneous detection of multiple DNA targets. J. Am. Chem. Soc..

[28] Kerman K., Saito M., Morita Y., Takamura Y., Ozsoz M., Tamiya E. (2004). Electrochemical coding of single-nucleotide polymorphisms by monobase-modified gold nanoparticles. Anal. Chem..

[29] Kerman K., Kobayashi M., Tamiya E. (2003). Recent trends in electrochemical DNA biosensor technology. Meas. Sci. Technol..

[30] Hernández-Santos D., Díaz-González M., González-Garcia M.B., Costa-García A. (2004). Enzymatic genosensor on streptavidin-modified screen-printed carbon electrodes. Anal. Chem..

[31] Wang J. (2002). Electrochemical nucleic acid biosensors. Anal. Chim. Acta.

[32] Paleček E., Fojta M., Tomschik M., Wang J. (1998). Electrochemical biosensors for DNA hybridization and DNA damage. Biosens. Bioelectron..

[33] Wang J. (2002). Electrochemical detection for microscale analytical systems: A review. Talanta.

[34] Chikae M., Idegami K., Kerman K., Nagatani N., Ishikawa M., Takamura Y., Tamiya E. (2006). Direct fabrication of catalytic metal nanoparticles onto the surface of a screen-printed carbon electrode. Electrochem. Commun..

[35] Idegami K., Chikae M., Kerman K., Nagatani N., Yuhi T., Endo T., Tamiya E. (2008). Gold nanoparticle-based redox signal enhancement for sensitive detection of human chorionic gonadotropin hormone. Electroanalysis Int. J. Devoted Fundam. Pract. Asp. Electroanal..

[36] Yuki K., Fujiogi M., Koutsogiannaki S. (2020). COVID-19 pathophysiology: A review. Clin. Immunol..

[37] Yamauchi Y., Helenius A. (2013). Virus entry at a glance. J. Cell Sci..

[38] Taubenberger J.K., Morens D.M. (2008). The pathology of influenza virus infections. Annu Rev. Pathol. Mech. Dis..

[39] Seitz R. (2016). Human Immunodeficiency Virus (HIV). Transfus. Med. Hemotherapy.

[40] Simon V., Ho D.D., Karim Q.A. (2006). HIV/AIDS epidemiology, pathogenesis, prevention, and treatment. Lancet.

[41] Domingo P., Mur I., Pomar V., Corominas H., Casademont J., de Benito N. (2020). The four horsemen of a viral Apocalypse: The pathogenesis of SARS-CoV-2 infection (COVID-19). EBioMedicine.

[42] Shang J., Wan Y., Luo C., Ye G., Geng Q., Auerbach A., Li F. (2020). Cell entry mechanisms of SARS-CoV-2. Proc. Natl. Acad. Sci. USA.

[43] El Ramahi R., Freifeld A. (2019). Epidemiology, diagnosis, treatment, and prevention of influenza infection in oncology patients. J. Oncol. Pract..

[44] Kalil A.C., Thomas P.G. (2019). Influenza virus-related critical illness: Pathophysiology and epidemiology. Crit. Care.

[45] Lv M., Luo X., Estill J., Liu Y., Ren M., Wang J., Wang Q., Zhao S., Wang X., Yang S. (2020). Coronavirus disease (COVID-19): A scoping review. Eurosurveillance.

[46] Rouse B.T., Sehrawat S. (2010). Immunity and immunopathology to viruses: What decides the outcome?. Nat. Rev. Immunol..

[47] Wang J. (2006). Electrochemical biosensors: Towards point-of-care cancer diagnostics. Biosens. Bioelectron..

[48] Katz E., Willner I. (2004). Biomolecule-functionalized carbon nanotubes: Applications in nanobioelectronics. ChemPhysChem.

[49] Kara P., Kerman K., Ozkan D., Meric B., Erdem A., Ozkan Z., Ozsoz M. (2002). Electrochemical genosensor for the detection of interaction between methylene blue and DNA. Electrochem. Commun..

[50] Alves R.D.F., Franco D.L., Cordeiro M.T., de Oliveira E.M., Dutra R.A.F., Sotomayor M.D.P.T. (2019). Novel electrochemical genosensor for Zika virus based on a poly-(3-amino-4-hydroxybenzoic acid)-modified pencil carbon graphite electrode. Sens. Actuators B Chem..

[51] Wang J. (2005). Carbon-Nanotube Based Electrochemical Biosensors: A Review. Electroanalysis.

[52] de Rooij D.M.R. (2003). Electrochemical Methods: Fundamentals and Applications. Anti-Corros. Methods Mater..

[53] Grieshaber D., MacKenzie R., Vörös J., Reimhult E. (2008). Electrochemical biosensors-sensor principles and architectures. Sensors.

[54] Singh A., Amin S.I., Anand S. (2020). Label free detection of biomolecules using SiGe sourced dual electrode doping-less dielectrically modulated tunnel FET. Silicon.

[55] Wadhera T., Kakkar D., Wadhwa G., Raj B. (2019). Recent advances and progress in development of the field effect transistor biosensor: A review. J. Electron. Mater..

[56] Lee D., Chander Y., Goyal S.M., Cui T. (2011). Carbon nanotube electric immunoassay for the detection of swine influenza virus H1nbiosens. Biosens. Bioelectron..

[57] Mahari S., Roberts A., Shahdeo D., Gandhi S. (2020). eCovSens-ultrasensitive novel in-house built printed circuit board based electrochemical device for rapid detection of nCovid-19 antigen, a spike protein domain 1 of SARS-CoV-2. bioRxiv.

[58] Chen Y., Ren R., Pu H., Guo X., Chang J., Zhou G., Mao S., Kron M.A., Chen J. (2017). Field-effect transistor biosensor for rapid detection of ebola antigen. Sci. Rep..

[59] Jin X., Zhang H., Li Y.-T., Xiao M.-M., Zhang Z., Pang D.-W., Wong G., Zhang G.-J. (2019). A field effect transistor modified with reduced graphene oxide for immunodetection of Ebola virus. Microchim. Acta.

[60] Benavente J. (2005). Electrochemical Impedance Spectroscopy as a Tool for Electrical and Structural Characterizations of Membranes in Contact with Electrolyte Solutions. Recent Advances in Multidisciplinary Applied Physics.

[61] Bertok T., Lorencova L., Chocholova E., Jane E., Vikartovska A., Kasak P., Tkac J. (2018). Electrochemical impedance spectroscopy based biosensors: Mechanistic principles, analytical examples and challenges towards commercialization for assays of protein cancer biomarkers. ChemElectroChem.

[62] Nakamura T., Homma K., Tachibana K. (2011). Impedance spectroscopy of manganite films prepared by metalorganic chemical vapor deposition. J. Nanosci. Nanotechnol..

[63] Vogt S., Su Q., Gutiérrez-Sánchez C., Nöll G. (2016). Critical view on electrochemical impedance spectroscopy using the ferri/ferrocyanide redox couple at gold electrodes. Anal. Chem..

[64] Xie N., Wang C., Lian Y., Wu C., Zhang H., Zhang Q. (2014). Inhibition of mitochondrial fission attenuates Aβ-induced microglia apoptosis. Neuroscience.

[65] Krammer F. (2019). The human antibody response to influenza a virus infection and vaccination. Nat. Rev. Immunol..

[66] Kiilerich-Pedersen K., Daprà J., Cherré S., Rozlosnik N. (2013). High sensitivity point-of-care device for direct virus diagnostics. Biosens. Bioelectron..

[67] Hai W., Goda T., Takeuchi H., Yamaoka S., Horiguchi Y., Matsumoto A., Miyahara Y. (2017). Specific recognition of human influenza virus with PEDOT bearing sialic acid-terminated trisaccharides. ACS Appl. Mater. Interfaces.

[68] Hai W., Goda T., Takeuchi H., Yamaoka S., Horiguchi Y., Matsumoto A., Miyahara Y. (2018). Human influenza virus detection using sialyllactose-functionalized organic electrochemical transistors. Sens. Actuators B Chem..

[69] Singh R., Hong S., Jang J. (2017). Label-free detection of influenza viruses using a reduced graphene oxide-based electrochemical immunosensor integrated with a microfluidic platform. Sci. Rep..

[70] Mantione D., del Agua I., Sanchez-Sanchez A., Mecerreyes D. (2017). Poly(3,4-ethylenedioxythiophene) (PEDOT) derivatives: Innovative conductive polymers for bioelectronics. Polymers.

[71] Strakosas X., Bongo M., Owens R.M. (2015). The organic electrochemical transistor for biological applications. J. Appl. Polym. Sci..

[72] Krejcova L., Nejdl L., Hynek D., Krizkova S., Kopel P., Adam V., Kizek R. (2013). Beads-based electrochemical assay for the detection of influenza hemagglutinin labeled with CdTe quantum dots. Molecules.

[73] Kamikawa T.L., Mikolajczyk M.G., Kennedy M., Zhang P., Wang W., Scott D.E., Alocilja E.C. (2010). Nanoparticle-based biosensor for the detection of emerging pandemic influenza strains. Biosens. Bioelectron..

[74] Ülkü A., Tepeli Y., Sayhi M., Nsiri J., Diouani M.F. (2018). Towards the electrochemical diagnostic of influenza virus: Development of a graphene–Au hybrid nanocomposite modified influenza virus biosensor based on neuraminidase activity. Analyst.

[75] Hassen W.M., Duplan V., Frost E., Dubowski J.J. (2011). Quantitation of influenza a virus in the presence of extraneous protein using electrochemical impedance spectroscopy. Electrochim. Acta.

[76] Sayhi M., Ouerghi O., Belgacem K., Arbi M., Tepeli Y., Ghram A., Ülkü A., Österlund L., Laouini D., Diouani M.F. (2018). Electrochemical detection of influenza virus H9N2 based on both immunomagnetic extraction and gold catalysis using an immobilization-free screen printed carbon microelectrode. Biosens. Bioelectron..

[77] Joshi S.R., Sharma A., Kim G.-H., Jang J. (2020). Low cost synthesis of reduced graphene oxide using biopolymer for influenza virus sensor. Mater. Sci. Eng. C.

[78] Tîlmaciu C.-M., Morris M.C. (2015). Carbon nanotube biosensors. Front. Chem..

[79] Mikuła E., Silva C.E., Kopera E., Zdanowski K., Radecki J., Radecka H. (2018). Highly sensitive electrochemical biosensor based on redox-active monolayer for detection of anti-hemagglutinin antibodies against swine-origin influenza virus H1N1 in sera of vaccinated mice. BMC Vet. Res..

[80] Jarocka U., Sawicka R., Stachyra A., Góra-Sochacka A., Sirko A., Zagórski-Ostoja W., Sączyńska V., Porebska A.J., Dehaen W., Radecki J. (2015). A biosensor based on electroactive dipyrromethene-Cu(II) layer deposited onto gold electrodes for the detection of antibodies against avian influenza virus type H5N1 in hen sera. Anal. Bioanal. Chem..

[81] Veerapandian M., Hunter R., Neethirajan S. (2016). Dual immunosensor based on methylene blue-electroadsorbed graphene oxide for rapid detection of the influenza a virus antigen. Talanta.

[82] Osteryoung J.G., Osteryoung R.A. (1985). Square wave voltammetry. Anal. Chem..

[83] Li Z.-N., Lin S.-C., Carney P.J., Li J., Liu F., Lu X., Liu M., Stevens J., Levine M., Katz J.M. (2014). IgM, IgG, and IgA antibody responses to influenza A(H1N1)pdm09 hemagglutinin in infected persons during the first wave of the 2009 pandemic in the United States. Clin. Vaccine Immunol..

[84] Padian N.S., Buvé A., Balkus J., Serwadda D., Cates W. (2008). Biomedical interventions to prevent HIV infection: Evidence, challenges, and way forward. Lancet.

[85] Urio L.J., Mohamed M.A., Mghamba J., Abade A.M., Aboud S. (2015). Evaluation of HIV antigen /antibody combination ELISA’s for diagnosis of HIV infection in Dar Es Salaam, Tanzania. Pan. Afr. Med. J..

[86] Ma Y., Shen X.-L., Zeng Q., Wang H.-S., Wang L.-S. (2017). A multi-walled carbon nanotubes based molecularly imprinted polymers electrochemical sensor for the sensitive determination of HIV-p24. Talanta.

[87] Fang Y.-S., Huang X.-J., Wang L.-S., Wang J.-F. (2015). An enhanced sensitive electrochemical immunosensor based on efficient encapsulation of enzyme in silica matrix for the detection of human immunodeficiency virus Pbiosens. Bioelectronics.

[88] Zhou L., Huang J., Yu B., Liu D., You T. (2015). A novel electrochemiluminescence immunosensor for the analysis of HIV-1 p24 antigen based on P-RGO@Au@Ru-SiO2 composite. ACS Appl. Mater. Interfaces.

[89] Giannetto M., Costantini M., Mattarozzi M., Careri M. (2017). Innovative gold-free carbon nanotube/chitosan-based competitive immunosensor for determination of HIV-related p24 capsid protein in serum. RSC Adv..

[90] Islam S., Shukla S., Bajpai V.K., Han Y.-K., Huh Y.S., Kumar A., Ghosh A., Gandhi S. (2019). A smart nanosensor for the detection of human immunodeficiency virus and associated cardiovascular and arthritis diseases using functionalized graphene-based transistors. Biosens. Bioelectron..

[91] Cerrutti B.M., Moraes M.L., Pulcinelli S.H., Santilli C.V. (2015). Lignin as immobilization matrix for HIV p17 peptide used in immunosensing. Biosens. Bioelectron..

[92] Fatin M., Ruslinda A.R., Gopinath S.C., Arshad M. (2019). High-performance interactive analysis of split aptamer and HIV-1 Tat on multiwall carbon nanotube-modified field-effect transistor. Int. J. Biol. Macromol..

[93] Majd S.M., Salimi A., Astinchap B. (2018). The development of radio frequency magnetron sputtered p-type nickel oxide thin film field-effect transistor device combined with nucleic acid probe for ultrasensitive label-free HIV-1 gene detection. Sens. Actuators B Chem..

[94] Yan Z., Gan N., Zhang H., Wang D., Qiao L., Cao Y., Li T., Huairong Z. (2015). A sandwich-hybridization assay for simultaneous determination of HIV and tuberculosis DNA targets based on signal amplification by quantum dots-PowerVision™ polymer coding nanotracers. Biosens. Bioelectron..

[95] Gong Q., Wang Y., Yang H. (2017). A sensitive impedimetric DNA biosensor for the determination of the HIV gene based on graphene-Nafion composite film. Biosens. Bioelectron..

[96] Gong Q., Yang H., Dong Y., Zhang W. (2015). A sensitive impedimetric DNA biosensor for the determination of the HIV gene based on electrochemically reduced graphene oxide. Anal. Methods.

[97] Wang Y., Bai X., Wen W., Zhang X., Wang S. (2015). Ultrasensitive electrochemical biosensor for HIV gene detection based on graphene stabilized gold nanoclusters with exonuclease amplification. ACS Appl. Mater. Interfaces.

[98] Yeter E.Ç., Şahin S., Caglayan M.O., Üstündağ Z. (2021). An electrochemical label-free DNA impedimetric sensor with AuNP-modified glass fiber/carbonaceous electrode for the detection of HIV-1 DNA. Chem. Pap..

[99] Teymourian H., Salimi A., Khezrian S. (2017). Development of a new label-free, indicator-free strategy toward ultrasensitive electrochemical DNA biosensing based on Fe_3_O_4_ nanoparticles/reduced graphene oxide composite. Electroanalysis.

[100] Li J. (2020). Ultrasensitive and highly selective electrochemical biosensor for HIV gene detection based on amino-reduced graphene oxide and β-cyclodextrin modified glassy carbon electrode. Int. J. Electrochem. Sci..

[101] Qiao J., Han H., Yang H., Zhang M., Sun X., Liang Y., Liu Z., Zhang W., Qiao J. (2019). Sensitive electrochemical DNA sensor for the detection of HIV based on a polyaniline/graphene nanocomposite. J. Mater..

[102] Shamsipur M., Samandari L., Taherpour A., Pashabadi A. (2019). Sub-femtomolar detection of HIV-1 gene using DNA immobilized on composite platform reinforced by a conductive polymer sandwiched between two nanostructured layers: A solid signal-amplification strategy. Anal. Chim. Acta.

[103] Rewar S., Mirdha D. (2015). Transmission of Ebola virus disease: An overview. Ann. Glob. Health.

[104] Malvy D., McElroy A.K., De Clerck H., Günther S., Van Griensven J. (2019). Ebola virus disease. Lancet.

[105] Yamanaka K., Vestergaard M.C., Tamiya E. (2016). Printable electrochemical biosensors: A focus on screen-printed electrodes and their application. Sensors.

[106] Ilkhani H., Farhad S. (2018). A novel electrochemical DNA biosensor for Ebola virus detection. Anal. Biochem..

[107] Carinelli S., Kühnemund M., Nilsson M., Pividori M. (2017). Yoctomole electrochemical genosensing of Ebola virus cDNA by rolling circle and circle to circle amplification. Biosens. Bioelectron..

[108] Demidov V.V. (2002). Rolling-circle amplification in DNA diagnostics: The power of simplicity. Expert Rev. Mol. Diagn..

[109] Chakhtoura N., Hazra R., Spong C.Y. (2018). Zika virus: A public health perspective. Curr. Opin. Obs. Gynecol..

[110] Agumadu V.C., Ramphul K. (2018). Zika virus: A review of literature. Asian Pac. J. Trop. Biomed..

[111] Noorbakhsh F., Abdolmohammadi K., Fatahi Y., Dalili H., Rasoolinejad M., Rezaei F., Salehi-Vaziri M., Shafiei-Jandaghi N.Z., Gooshki E.S., Zaim M. (2019). Zika virus infection, basic and clinical aspects: A review article. Iran. J. Public Health.

[112] Gourinat A.-C., O’Connor O., Calvez E., Goarant C., Dupont-Rouzeyrol M. (2015). Detection of zika virus in urine. Emerg. Infect. Dis..

[113] Musso D., Roche C., Nhan T.-X., Robin E., Teissier A., Cao-Lormeau V.-M. (2015). Detection of zika virus in saliva. J. Clin. Virol..

[114] Liuzzi G., Nicastri E., Puro V., Zumla A., Ippolito G. (2016). Zika virus in saliva—New challenges for prevention of human to human transmission. Eur. J. Intern. Med..

[115] Faye O., Faye O., Dupressoir A., Weidmann M., Ndiaye M., Sall A.A. (2008). One-step RT-PCR for detection of Zika virus. J. Clin. Virol..

[116] Vorou R. (2016). Letter to the editor: Diagnostic challenges to be considered regarding Zika virus in the context of the presence of the vector Aedes albopictus in Europe. Eurosurveillance.

[117] Nicolini A.M., McCracken K.E., Yoon J.-Y. (2017). Future developments in biosensors for field-ready Zika virus diagnostics. J. Biol. Eng..

[118] Lynch C.A., Foguel M.V., Reed A.J., Balcarcel A.M., Calvo-Marzal P., Gerasimova Y.V., Chumbimuni-Torres K.Y. (2019). Selective determination of isothermally amplified Zika Virus RNA using a universal DNA-hairpin probe in less than 1 h. Anal. Chem..

[119] Mills D.M., Foguel M.V., Martin C.P., Trieu T.T., Kamar O., Calvo-Marzal P., Kolpashchikov D.M., Chumbimuni-Torres K.Y. (2019). Rapid detection of different DNA analytes using a single electrochemical sensor. Sens. Actuators B Chem..

[120] Zhang Y.-W., Liu W.-S., Chen J.-S., Niu H.-L., Mao C.-J., Jin B.-K. (2020). Metal-organic gel and metal-organic framework based switchable electrochemiluminescence RNA sensing platform for Zika virus. Sens. Actuators B Chem..

[121] Compton J. (1991). Nucleic acid sequence-based amplification. Nat. Cell Biol..

[122] Cabral-Miranda G., Cardoso A.R., Ferreira L.C., Sales M.G.F., Bachmann M.F. (2018). Biosensor-based selective detection of Zika virus specific antibodies in infected individuals. Biosens. Bioelectron..

[123] Priyamvada L., Hudson W., Ahmed R., Wrammert J. (2017). Humoral cross-reactivity between Zika and dengue viruses: Impli-cations for protection and pathology. Emerg. Microbes Infect..

[124] Reyes-Sandoval A., Ludert J.E. (2019). The dual role of the antibody response against the flavivirus non-structural protein 1 (NS1) in protection and immuno-pathogenesis. Front. Immunol..

[125] Auer G.K., Oliver P.M., Rajendram M., Lin T.-Y., Yao Q., Jensen G.J., Weibel D.B. (2019). Bacterial swarming reducesproteus mirabilisandVibrio parahaemolyticusCell stiffness and increases β-lactam susceptibility. mBio.

[126] Afsahi S., Lerner M.B., Goldstein J.M., Lee J., Tang X., Bagarozzi D.A., Pan D., Locascio L., Walker A., Barron F. (2018). Novel graphene-based biosensor for early detection of Zika virus infection. Biosens. Bioelectron..

[127] Faria A.M., Mazon T. (2019). Early diagnosis of Zika infection using a ZnO nanostructures-based rapid electrochemical biosensor. Talanta.

[128] Kaushik A., Yndart A., Kumar S., Jayant R.D., Vashist A., Brown A.N., Li C.-Z., Nair M. (2018). A sensitive electrochemical immunosensor for label-free detection of Zika-virus protein. Sci. Rep..

[129] Tancharoen C., Sukjee W., Thepparit C., Jaimipuk T., Auewarakul P., Thitithanyanont A., Sangma C. (2018). Electrochemical biosensor based on surface imprinting for Zika virus detection in serum. ACS Sens..

[130] Kaushik A., Tiwari S., Jayant R.D., Vashist A., Nikkhah-Moshaie R., El-Hage N., Nair M.P.N. (2017). Electrochemical biosensors for early stage Zika diagnostics. Trends Biotechnol..

[131] Shim E., Tariq A., Choi W., Lee Y., Chowell G. (2020). Transmission potential and severity of COVID-19 in South Korea. Int. J. Infect. Dis..

[132] Kilic T., Weissleder R., Lee H. (2020). Molecular and immunological diagnostic tests of COVID-19: Current status and challenges. Science.

[133] Vashist S.K. (2020). In Vitro diagnostic assays for COVID-19: Recent advances and emerging trends. Diagnostics.

[134] Lupia T., Scabini S., Pinna S.M., Di Perri G., De Rosa F.G., Corcione S. (2020). 2019 novel coronavirus (2019-nCoV) outbreak: A new challenge. J. Glob. Antimicrob. Resist..

[135] Corman V.M., Landt O., Kaiser M., Molenkamp R., Meijer A., Chu D.K., Bleicker T., Brünink S., Schneider J., Schmidt M.L. (2020). Detection of 2019 novel coronavirus (2019-nCoV) by real-time RT-PCR. Eurosurveillance.

[136] Nayak S., Blumenfeld N.R., Laksanasopin T., Sia S.K. (2017). Point-of-care diagnostics: Recent developments in a connected age. Anal. Chem..

[137] Choi J.R. (2020). Development of point-of-care biosensors for COVID-19. Front. Chem..

[138] Tripathy S., Singh S.G. (2020). Label-free electrochemical detection of DNA hybridization: A method for COVID-19 diagnosis. Trans. Indian Natl. Acad. Eng..

[139] Chan J.F.-W., Yip C.C.-Y., To K.K.-W., Tang T.H.-C., Wong S.C.-Y., Leung K.-H., Fung A.Y.-F., Ng A.C.-K., Zou Z., Tsoi H.-W. (2020). Improved molecular diagnosis of COVID-19 by the novel, highly sensitive and specific COVID-19-RdRp/Hel real-time reverse transcription-PCR assay validated In Vitro and with clinical specimens. J. Clin. Microbiol..

[140] Seo G., Lee G., Kim M.J., Baek S.-H., Choi M., Ku K.B., Lee C.-S., Jun S., Park D., Kim H.G. (2020). Rapid detection of COVID-19 causative virus (SARS-CoV-2) in human nasopharyngeal swab specimens using field-effect transistor-based biosensor. ACS Nano.

[141] Nadoushan M.J., Ahmadi S., Nadoushan P.J. (2020). Serology testing for SARS-CoV-2: Benefits and challenges. Iran. J. Pathol..

[142] Tan W., Lu Y., Zhang J., Wang J., Dan Y., Tan Z., He X., Qian C., Sun Q., Hu Q. (2020). Viral kinetics and antibody responses in patients with COVID-19. medRxiv.

[143] Mavrikou S., Moschopoulou G., Tsekouras V., Kintzios S. (2020). Development of a portable, ultra-rapid and ultra-sensitive cell-based biosensor for the direct detection of the SARS-CoV-2 S1 spike protein antigen. Sensors.

[144] Raziq A., Kidakova A., Boroznjak R., Reut J., Öpik A., Syritski V. (2021). Development of a portable MIP-based electrochemical sensor for detection of SARS-CoV-2 antigen. Biosens. Bioelectron..

[145] Zhao H., Liu F., Xie W., Zhou T.-C., Ouyang J., Jin L., Li H., Zhao C.-Y., Zhang L., Wei J. (2021). Ultrasensitive supersandwich-type electrochemical sensor for SARS-CoV-2 from the infected COVID-19 patients using a smartphone. Sens. Actuators B Chem..

[146] Huang L., Ding L., Zhou J., Chen S., Chen F., Zhao C., Xu J., Hu W., Ji J., Xu H. (2021). One-step rapid quantification of SARS-CoV-2 virus particles via low-cost nanoplasmonic sensors in generic microplate reader and point-of-care device. Biosens. Bioelectron..

[147] Cady N.C., Tokranova N., Minor A., Nikvand N., Strle K., Lee W.T., Page W., Guignon E., Pilar A., Gibson G.N. (2021). Multiplexed detection and quantification of human antibody response to COVID-19 infection using a plasmon enhanced biosensor platform. Biosens. Bioelectron..

[148] Fabiani L., Saroglia M., Galatà G., De Santis R., Fillo S., Luca V., Faggioni G., D’Amore N., Regalbuto E., Salvatori P. (2021). Magnetic beads combined with carbon black-based screen-printed electrodes for COVID-19: A reliable and miniaturized electrochemical immunosensor for SARS-CoV-2 detection in saliva. Biosens. Bioelectron..

[149] Rashed M.Z., Kopechek J.A., Priddy M.C., Hamorsky K.T., Palmer K.E., Mittal N., Valdez J., Flynn J., Williams S.J. (2021). Rapid detection of SARS-CoV-2 antibodies using electrochemical impedance-based detector. Biosens. Bioelectron..

